# Molecular Species Identification and Genotyping of Free-Living Amoebae in Soil of Recreational Mountain Areas in the Babiogórski National Park and Surroundings, Southern Poland

**DOI:** 10.3390/ijms26178160

**Published:** 2025-08-22

**Authors:** Małgorzata Adamska

**Affiliations:** Department of Genetics and Genomics, Institute of Biology, University of Szczecin, Felczaka 3c, 71-412 Szczecin, Poland; malgorzata.adamska@usz.edu.pl

**Keywords:** free-living amoebae, soil, *SSU rRNA*, genotyping, thermotolerance

## Abstract

Free-living amoebae (FLAs) are widely present in the environment and may be pathogenic for animals and humans. Studies on the prevalence of FLAs in European soils are few in number. This study aimed to molecularly identify the species and genotypes of FLAs occurring in soil from Southern Poland. Forty soil samples were collected in June 2024 in the Babiogórski National Park. Amoebae cultures and a thermal-tolerance test were conducted, and all strains grew at 37 °C. Five PCR protocols were applied for the amplification of FLA *SSU rRNA* fragments. The following FLAs, including potentially pathogenic ones, were detected: *Acanthamoeba* T4 and T13 genotypes in 79.1% of positive samples, *Naegleria gruberi* and *Naegleria galeacystis* in 25%, *Vermamoeba vermiformis* in 12.5%, and *Paravahlkampfia* sp. and *Ptolemeba bulliensis* in 8.3%. Species and genotype identification were determined by sequence comparison and phylogenetic analysis. This study reports, for the first time, the isolation of *N. galeacystis* from soil and *N. gruberi* and *V*. *vermiformis* from soil collected in Europe. The used primer sets have different usefulness for *Naegleria* species identification and their phylogenetic analysis. The primers applied in this study may not reveal the full diversity of amoebae in soil; therefore, it is necessary to design new primers for this purpose.

## 1. Introduction

Free-living amoebae (FLAs) are a polyphyletic group of protists that belong to three supergroups within the kingdom Protozoa: Amoebozoa, e.g., *Acanthamoeba* spp., *Vermamoeba vermiformis* (formerly *Hartmannella vermiformis*), *Balamuthia* spp., and *Sappinia* spp.; Excavata, e.g., *Naegleria* spp.; and Rhizaria, e.g., *Paulinella* [1,2,3]. FLAs can survive and reproduce in the environment, and depending on external conditions, they may occur as active, feeding trophozoites or dormant, resistant cysts. They play critical ecological roles and interact with other microorganisms, forming a predator-prey, symbiotic, or host–parasite relationship with bacteria. FLAs may serve as hosts for many animal and human pathogens, contributing to their survival, spread, and transmission [1,4,5]. Some free-living amoebae are pathogenic and may cause infections of the central nervous system (*Acanthamoeba* spp., *Balamuthia mandrillaris*, *Sappinia pedata*, and *Naegleria fowlerii*) or keratitis (*Acanthamoeba* spp. and *Vermamoeba vermiformis*) [2,3,5,6,7]. A few authors suggest the possibility of FLA presence in the gut microbiome of mammals and other animals [8].

Some FLAs infecting humans have been well genetically analyzed and divided into genotypes. Some of them are potentially pathogenic, while the remaining genotypes have not been associated with infection to date. Based on *18S rRNA* gene sequence analysis, 23 genotypes (T1 to T23) of *Acanthamoeba* species have been distinguished. Some genotypes cause granulomatous amoebic encephalitis (GAE), *Acanthamoeba* keratitis, or both. In the case of *N. fowlerii*, five of the eight genotypes, established based on the sequence of ITS1 and *5.8S rRNA* gene, have been isolated from clinical cases of primary amoebic meningoencephalitis (PAM) [2]. There is no genotype classification for *B. mandrillaris* due to a lack of or low variation in its *18S rRNA* and mitochondrial *16S rRNA* genes, respectively [2]. The pathogenicity of *V. vermiformis* is questionable, and limited molecular analyses of this species demonstrated a low diversity of its *18S rRNA* gene. However, the latest research, based on multi-locus analysis, revealed a high degree of genetic diversity and the presence of multiple cryptic species within *V. vermiformis* [2,9]. *S. pedata* is linked with only one case of encephalitis. The sequences of the *18S rRNA* gene and ITS1, *5.8S*, and ITS2 regions of the four analyzed isolates demonstrated a size variation, but little is known about the genetic diversity of this species [2]. In the case of *Acanthamoeba*, thermotolerance and osmotolerance are considered indicators of the strain’s pathogenicity potential. However, further studies are required to clarify this question [10,11].

FLAs are widely present in various natural and artificial environments and have been isolated from different water sources, solid matrices, and air [5,12]. Many studies have been conducted on the prevalence and genetic diversity of FLAs in various solid matrices, including soil, mud, sand, sediments, compost, and dust [12]. However, there are few European studies on this topic, especially regarding non-*Acanthamoeba* FLAs. Almost all previous studies were based on amoebae cultures before their identification. Using environmental DNA (eDNA), total DNA isolated from environmental samples (e.g., soil) originating from various organisms, helps reduce the time needed for examinations. It also allows for the detection of more species from the same sample. The challenge of the eDNA approach is the presence of PCR inhibitors or the abundance of different organisms in the same sample [13,14], which can lead to false-negative or false-positive results, respectively.

This study aims to identify potentially pathogenic FLA species occurring in soil in recreational mountain areas in Southern Poland, using PCR and sequencing, their genotyping, and verification of thermal tolerance. The next aim is to compare the usefulness of eDNA and DNA isolated from amoebae cultures for PCR amplification of FLA *18S rRNA* gene fragments. The purpose is also to compare the sensitivity and specificity of five PCR protocols for FLA detection. The next aim was to evaluate the usefulness of the obtained *SSU rRNA* gene fragments for species identification, genotyping, and phylogenetic analysis. The knowledge regarding the prevalence, genetic diversity, and phylogenetic relationship of the detected amoebae will shed light on their biogeography and ecology and help evaluate their pathogenic potential and the health threat to visitors in the analyzed area.

## 2. Results

### 2.1. Results of Amoebae Cultures, Thermal Tolerance Test, PCRs, and Sequencing

PCR with JDP1/JDP2 primers and eDNA isolated directly from soil yielded a negative result. Using other primers, only non-specific products were obtained with eDNA.

Among all 40 soil samples cultured at 28 °C, FLAs were observed under a microscope in 24 cases (60%). After incubation of the transferred trophozoites at 37 °C and 42 °C, FLAs were observed, respectively, on 24 plates (60% of all samples, 100% of all observed FLAs) and 12 plates (30% of all samples, 50% of all observed FLAs). All the FLAs that grew at 37 °C were washed off the plates, and DNA was extracted from the rinsed material. FLAs that grew at 42 °C were not rinsed for DNA isolation, as only a few trophozoites were observed on the surface of individual agar plates. Next, PCR reactions were performed to detect FLAs. The *Acanthamoeba* genus was detected using the JDP1/JDP2 primer set in 19 of 24 (79.1%) DNA samples. The *Naegleria* genus was detected using the FLA-F/FLA-R, Ami6F1/Ami9R, and AmeF977/AmeR1534 primer sets in 6/24 (25%) DNA samples. *V*. *vermiformis* was detected using the FLA-F/FLA-R, AmeF977/AmeR1534, and HARTfor/HARTrev primer sets in 3/24 (12.5%) DNA samples. Other amoebae (*Paravahlkampfia* sp. and *Ptolemeba bulliensis*) were detected using the FLA-F/FLA-R and AmeF977/AmeR1534 primer sets, respectively, in 2/24 (8.3%) DNA samples. Co-occurrence of two different amoebae was detected in 7/24 (29.16%) of the DNA samples. Among all *Acanthamoeba* strains, 63.2% (12/19) represent the T4 genotype, and 36.8% (7/19) the T13 genotype. The detailed results of amoebae cultures at 42 °C, PCRs with individual primer pairs, and sequencing are presented in Table 1.

### 2.2. Results of Sequence Comparison and Phylogenetic Analysis

**Figure 1:**
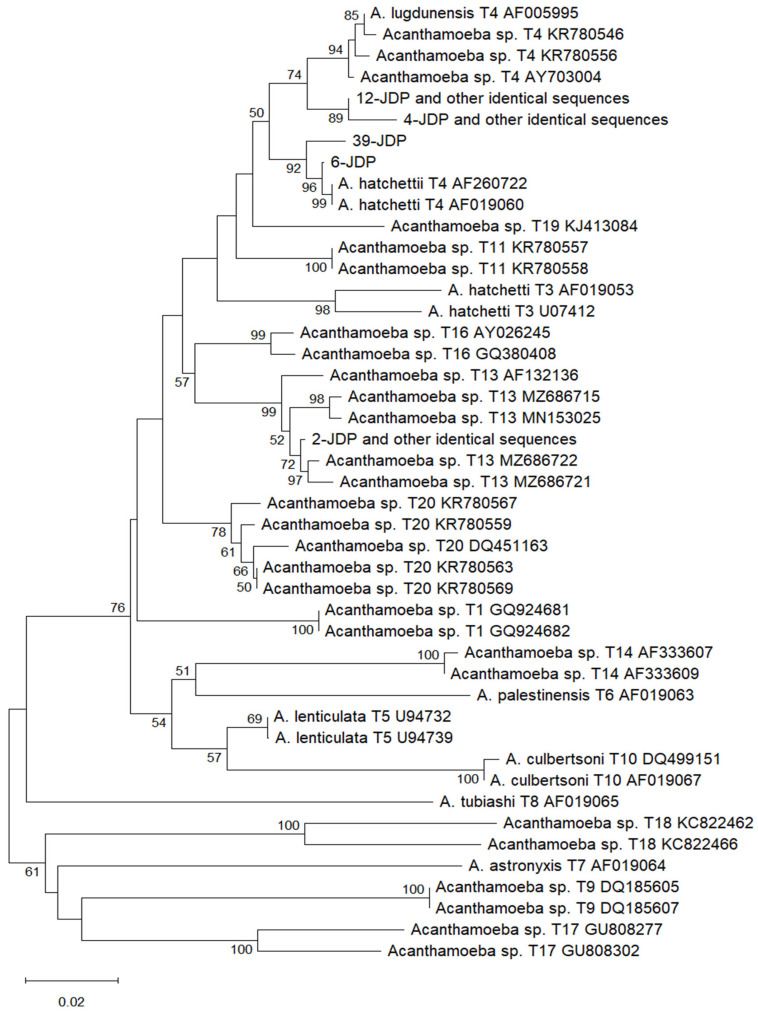
Phylogenetic tree constructed using the neighbor-joining statistical method and the Kimura 2-parameter model with 1000 bootstrap sampling, based on forty-five *SSU rRNA* sequences of *Acanthamoeba*. The forty sequences with accession numbers are from GenBank, and the remaining five sequences were obtained in this study using the JDP1/JDP2 primer set. Identical sequences are counted as a single sequence. Only bootstrap values ≥ 50 are shown.

**Figure 2:**
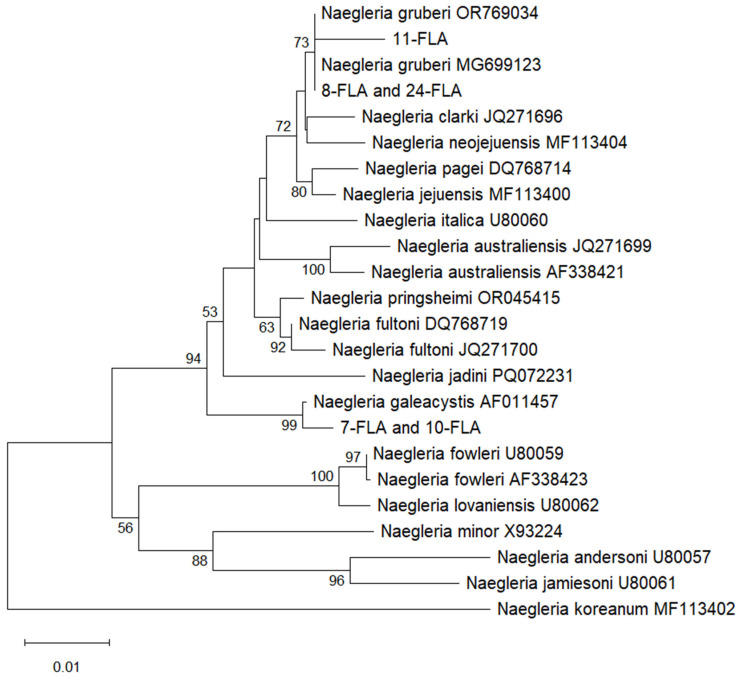
A phylogenetic tree constructed using the neighbor-joining statistical method and the Kimura 2-parameter model with 1000 bootstrap sampling, based on twenty-four *SSU rRNA* sequences of the *Naegleria* genus. Twenty-one sequences with accession numbers are from GenBank, and the remaining three sequences were obtained in this study using the FLA-F/FLA-R primer set. Identical sequences are counted as a single sequence. Only bootstrap values ≥ 50 are shown.

The *Acanthamoeba* genotype and the species of the remaining amoebae were identified by aligning their sequences with those in the GenBank database: (https://www.ncbi.nlm.nih.gov/genbank/, accessed on 19 May 2025) using the BLAST tool: https://blast.ncbi.nlm.nih.gov/Blast.cgi (accessed on 19 May 2025), as well as by analyzing the topology of the phylogenetic trees (Figure 1 and Figure 2).

Twelve of nineteen *Acanthamoeba* sequences obtained in this study have shown 100% identity to the T4 sequences previously published in the Genbank database (samples 12, 15, 19, 27, 29, and 31 to the sequence ON408415; samples 4, 21, 37, and 40 to the sequences JQ669659, KT892868, MN700280, and MT378239; sample 6 to the sequence KT985967; and sample 39 to the sequence AF019060). The remaining seven *Acanthamoeba* sequences, obtained from samples 2, 10, 23, 28, 32, 33, and 35, were identical to each other and showed 99.48% identity to the T13 sequence MZ686722 from GenBank. The *Naegleria* sequences obtained from samples 7 and 10 were identical to each other and to the sequences DQ768717 and PP174311 of *Naegleria* sp. (FLA-F/FLA-R primers) or the sequence AF011457 of *N. galeacystis* (AmeF977/Ame R1534 primers). The identical sequences, obtained from samples 8 and 24 using FLA-F/FLA-R and Ami6F1/Ami9R primers, showed 100% identity to OR769034, MG699123, and AB298288 sequences of *N. gruberi*. The sequences obtained from sample 11 showed 98.64% (FLA-F/FLA-R primers) or 99.17% (Ami6F1/Ami9R primers) identity to the *N. gruberi* sequence mentioned above. Three identical sequences, obtained from samples 1, 11, and 24 using AmeF977/Ame R1534 primers, showed 100% identity to different *Naegleria* species: *N. gruberi* (OR769034, MG699123, AB298288), *N. clarki* (AF338417, AF338419, JQ271691-92, JQ271697, JQ271704-05), and *N. pringsheimi* (OR045415). All five obtained sequences of *V. vermiformis* were identical to the sequences from the GenBank database. Three of them, obtained from samples 2, 19, and 32 using the HARTfor/HARTrev primer pair, were identical to each other. The sequence obtained from sample 4 using the FLA-F/FLA-R primer set shows 99.25% identity to the sequence DQ388521 from the GenBank database, representing *Paravahlkampfia* sp. The sequence obtained from sample 19 using the AmeF977/AmeR1534 primer set shows 98.94% identity to the sequence PP732398 from the GenBank database, representing *Ptolemeba bulliensis* isolated from water (Philippines).

The phylogenetic trees based on the sequences obtained in this study and derived from the GenBank database are presented in Figure 1 and Figure 2. The twelve *Acanthamoeba* sequences (samples 4, 6, 12, 15, 19, 21, 27, 29, 31, 37, 39, and 40) obtained in this study belong to a clade consisting of T4 genotype sequences from the GenBank database. The seven sequences (samples 2, 10, 23, 28, 32, 33, and 35) belong to a clade that contains T13 genotype sequences from GenBank (Figure 1). These results are consistent with the results of sequence comparison. In the case of the *Naegleria* genus, the tree based on the products of the FLA-F/FLA-R primer set was the most reliable and is presented in Figure 2. The three sequences of *Naegleria* obtained in this study (samples 8, 11, 24) make a group with the *N. gruberi* sequences from GenBank, while the two sequences (samples 7 and 10) are grouped with the *N. galeacystis* sequence from GenBank. These results are consistent with the results of sequence comparison.

### 2.3. Results of Statistical Analysis

Statistical analysis shows significant differences in the prevalence of genera *Acanthamoeba* and *Naegleria*, *Acanthamoeba* genus and *Vermamoeba vermiformis*, and *Acanthamoeba* genus and other amoebae (*Paravahlkampfia* sp. and *Ptolemeba bulliensis*). The *p*-values were 0.0262, 0.314, and 0.0338, respectively. The differences between *Naegleria* genus, *V. vermiformis*, and other amoebae, as well as between T4 and T13 genotypes of *Acanthamoeba,* are not statistically significant. The analysis does not show statistically significant differences in the prevalence of different amoebae between the samples collected in the particular zones.

## 3. Discussion

Studies on FLA prevalence in the environment include cultures of amoebae, which increase their number and reduce the amount of inhibitors. However, this approach is time-consuming and allows the detection of only those amoebae that can be grown under the used conditions. In this study, an attempt was made to molecularly detect FLAs in eDNA isolates obtained directly from the soil. The use of a kit intended for DNA isolation from soil enabled the effective removal of inhibitors, as PCR products were obtained from eDNA using all primer pairs except the JDP1/JDP2 pair. However, all obtained products were nonspecific and derived from non-amoebae organisms. They may be more numerous in soil than amoebae, and FLA-intended primers may nonspecifically hybridize with their DNA, resulting in amplification of nonspecific products. Using DNA isolates obtained from amoebae cultures, specific products were obtained with all primer pairs. The *Acanthamoeba* genus-specific primer set, JDP1/JDP2, was the most specific of all used as it did not amplify non-specific products. However, it was not sensitive enough to amplify the DNA of amoebae isolated directly from soil. Cultures of amoebae from soil samples and their passage during a thermal-tolerance test contribute to increasing their numbers and removing most other soil organisms, which decreases the risk of false-negative and false-positive results. Amoebae cultures are necessary to detect these organisms in soil samples using the primers applied in this study.

The primer pairs used in this study have been applied in other papers for FLA detection in cultures from soil samples [15,16,17,18,19]. However, the authors did not compare their sensitivity and specificity. In this study, FLA-F/FLA-R and AmeF977/AmeR1534 sets appeared to be the most sensitive among the primers intended for different FLA DNA amplification. They both amplified the amoebae DNA in seven samples. However, in one sample, *Paravahlkampfia* sp. was detected only with the FLA-F/FLA-R set, and in another two samples, *Ptolemeba bulliensis* and *Naegleria* sp. were detected only with the AmeF977/AmeR1534 set. The Ami6F1/Ami9R set was the least sensitive and allowed for the detection of *N. gruberi* in the three samples in which this species was detected using the FLA-F/FLA-R set. The cause of the different sensitivity of the FLA-intended primers used in this study may be the varying grade of polymorphism of the *Naegleria* sequence at the sites of the primers’ hybridization, which influences the effectiveness of their binding to DNA. The FLA-F/FLA-R set seems to be the best choice for FLA detection and species identification, but it was not sensitive enough to amplify amoebae DNA in the two samples positive with the AmeF977/AmeR1534 set. None of the primer pairs intended for different FLAs allowed for the detection of *Acanthamoeba*, although theoretically they should hybridize with its DNA. The reason for the lack of PCR products for *Acanthamoeba* may be the sequence variability at the primer binding sites and a lack of primer hybridization to the DNA of the strains detected in this study. Reyes-Batlle et al. [20] applied the FLA-F/FLA-R primer set for *Acanthamoeba* detection in 24 soil samples from El Hierro Island (Canary Islands). They also failed to detect *Acanthamoeba* in any of the examined samples, despite this species being one of the most common in soil and occurring in the Canary Islands [21]. They identified only *V. vermiformis* in 20.8% of the samples. In this study, *V*. *vermiformis* was detected in 12.5% of culture-positive samples, in only one sample using FLA-F/FLA-R and AmeF977/AmeR1534 pairs, in contrast to three samples with the use of the HARTfor/HARTrev set, which is intended for the detection of the former genus *Hartmannella*. The application of the primers for a wide range of FLAs used in this study does not reveal the full diversity of soil amoebae. It is necessary to design new, sensitive primers enabling the detection and species identification of FLAs in soil and other environmental samples. A major challenge would be to create these primers so that their sensitivity and specificity are sufficient to detect FLAs in eDNA isolated directly from soil. The use of costly metagenomics techniques would provide complete knowledge of the amoebae diversity in soil.

Studies on the prevalence of a wide range of FLAs in soil and their genotyping are few in Europe. In Poland, Hendiger-Rizo et al. [16] detected *Acanthamoeba* spp. in most of the soil samples (78.2%) from Warsaw parks and squares, followed by *Platyamoeba placida*, *Stenamoeba berchidia*, and *Allovahlkampfia* sp. Denet et al. [22] examined the diversity of culturable FLAs at the genus level in French alpine soils, based on PCR and morphological analysis. They revealed that *Acanthamoeba* was the dominant genus (77%) among all detected FLAs, and the remaining detected amoebae belonged to *Tetramitus* and *Stachyamoeba* genera, or were unidentified. In this study, *Acanthamoeba* sp. was also detected in the majority of positive cultures (79.1%) and occurred significantly more frequently than other identified amoebae: *Naegleria* spp., *H. vermiformis*, *P*. *bulliensis*, and *Paravahlkampfia* sp. The differences between this study and the others may result from distinct climate or soil properties, or, in the case of the French study, may be caused by methodological differences. Other studies on the presence of different amoebae in soil used samples from outside Europe. In samples from Turkey, Iran, Tenerife, Santiago Island of Cape Verde, and the Canary Islands, *Acanthamoeba* sp. was the most prevalent FLA [17,18,19,21,23,24]. *V. vermiformis* was the most prevalent FLA in Northern Iran, Bolivia, and Guadeloupe [25,26,27]. *Naegleria* and *Tetramitus* genera dominated in Vietnam and Burkina Faso, respectively [22]. *Acanthamoeba* sp. seems to be a dominant genus in European soils, and there are considerable differences in FLA composition between sampling areas worldwide. Additional studies on the prevalence and distribution of FLAs in soil are necessary, and this research provides valuable insights into the subject. *Acanthamoeba* spp., *Naegleria* spp., and *V*. *vermiformis* have frequently been found in soil [9,12,15,17,18,19,20,23,24,25,28]. However, the Hartmannellid *genus Ptolemeba* and the Valkhampfiidae amoebae *Paravahlkampfia* sp. and *Naegleria* other than *N. fowleri* have been rarely detected in environmental samples. *Ptolemeba bulliensis* and *Ptolemeba noxubium* have been isolated from soil and water in Mississippi, respectively [29]. *P. bulliensis* has also been found in the gills of rainbow trout (*Oncorhynchus mykiss*) in Russia [30] and in water from the Philippines (PP732398, GenBank, unpublished). *Paravahlkampfia lenta* has been isolated from soil samples collected from a Scottish farm [31], and *Paravahlkampfia ustiana* from soil samples collected in Thailand [28]. *N. gruberi* has been isolated from soil samples from the UK and California [32]. This study is the first to reveal the presence of *N. galeacystis* in soil, as well as *N. gruberi* and *V. vermiformis* in soil from Europe.

Studies on the prevalence of *Acanthamoeba* genotypes in European soils were conducted in Poland, Austria, The Netherlands, Hungary, and Sardinia. The composition of *Acanthamoeba* genotypes was different depending on the examined area. The T4 genotype was the only one detected in the soil [16] and sandboxes [33] in Poland, as well as in rhizosphere samples from Hungary [34]. This genotype dominated in soils from Austria [35] and Sardinia [36], and accounted for half of all the detected *Acanthamoeba* strains in soil from The Netherlands [36]. Additionally, the T2 genotype has been found in soil from Austria and The Netherlands [35,36], T16 in soil from The Netherlands, and T13 in soil from Sardinia [36]. In this study, the T4 and T13 genotypes were identified. The first one dominated; however, the difference was not statistically significant. The T4 genotype was the most common in the European soil samples examined so far, and the share of the remaining genotypes was different in each examined area. Additional studies are necessary to investigate whether T4 predominance is typical in European soils and whether other genotypes, not detected so far, occur. The share of FLA species and *Acanthamoeba* genotypes differed even between relatively close locations, such as central [16,33] and southern Poland (this study). The composition of FLAs in soil, therefore, appears to be influenced by local conditions, such as soil properties. However, in this study, there were no significant differences in the occurrence of individual FLAs in different zones of the Babiogórski National Park.

Both *Acanthamoeba* genotypes identified in this study and *V*. *vermiformis* can cause keratitis, and the T4 *Acanthamoeba* genotype is an etiological agent of granulomatous amoebic encephalitis [6,37]. The *Paravahlkampfia* genus was previously considered a human pathogen [38,39,40]. The presence of these amoebae in the examined samples may indicate a potential health threat to individuals visiting the studied area. Tourists frequently visit the Babiogórski National Park, and maintaining hygiene, such as hand washing, is difficult in this location, which increases the risk of infection. Thermal-tolerant *Acanthamoeba* strains are considered potentially pathogenic [10,11]. All *Acanthamoeba* strains detected in this study were grown at temperatures of 28 °C and 37 °C, and numerous clusters of trophozoites were observed. Half of the T4 strains (6/12) and 42,8% of the T13 strains (3/7) were grown at a temperature of 42 °C, but only a few trophozoites were observed after incubation at this temperature. All strains of *Acanthamoeba* detected in this study are potentially pathogenic, as their DNA was isolated from strains that grew abundantly at 37 °C. However, the study of Kahraman et al. [10] suggests that thermotolerance does not indicate the pathogenicity of *Acanthamoeba*.

## 4. Materials and Methods

### 4.1. Study Area and Soil Sampling

Babia Góra National Park is situated in the Western Carpathians and is a popular destination for tourists. Babia Góra is the highest peak in the park (1725 m above sea level), and mixed forests cover most of the park area. The main tree species growing in the park are common beech (*Fagus sylvatica*), Norway spruce (*Picea abies*), and silver fir (*Abies alba*). Fir and beech forests, including Norway spruce and sycamore (*Acer pseudoplatanus*), dominate the lower montane zone (up to 1150 m). Norway spruce grows in the upper montane zone (up to 1390 m) with an admixture of rowan (*Sorbus aucuparia*). The next zone (up to 1650 m) is dominated by dwarf mountain pine (*Pinus mugo*). However, other species, such as dwarf forms of spruce, rowan, Silesian willow (*Salix silesiaca*), rock currant (*Ribes petraeum*), and mountain juniper (*Juniperus communis* subsp. *alpina*) are also present. Only low grasslands, mosses, and lichens grow in the Alpine zone (up to 1725 m).

**Figure 3:**
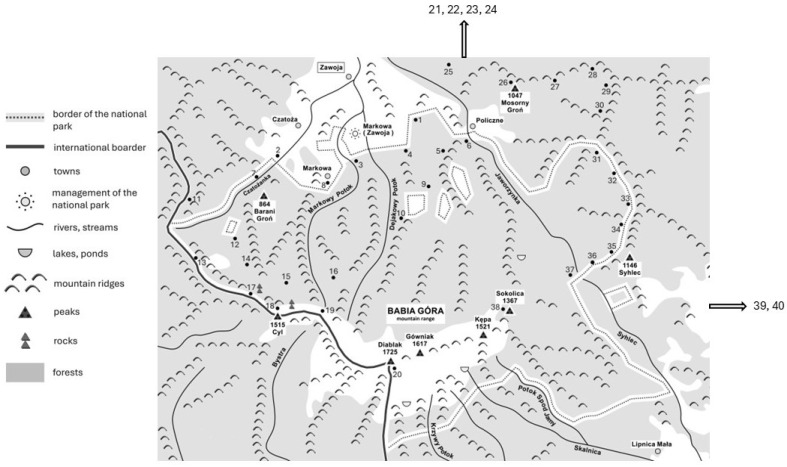
The collection sites of soil samples in the Babiogórski National Park and its surroundings. Numbers 21–24, 39, and 40 are the collection sites outside the national park.

Forty soil samples (approximately 5.0 mL each) were collected in June 2024 from Babia Góra National Park and its surrounding areas in southern Poland (Figure 3). Thirty-two samples (1–15, 21–28, 31–37, 39, and 40) were taken from the lower montane zone, four samples (16, 29, 30, 38) from the upper montane zone, three samples (17–19) from the zone of dwarf mountain pine, and one sample (20) from the Alpine zone. The samples were taken directly from the ground surface along marked tourist trails. During the sampling, the air temperature oscillated between 15 and 20 °C. The obtained material was placed in sterile Eppendorf tubes (Eppendorf, Hamburg, Germany) and stored at 4 °C until further processing and analysis in the laboratory.

### 4.2. Amoebae Cultures and Thermal Tolerance Test

In total, 1 g of each soil sample was seeded onto a Petri dish with NN-agar (A&A Biotechnology, Gdańsk, Poland) coated with heat-killed *Escherichia coli* (Hirszfeld Institute of Immunology and Experimental Therapy, Polish Academy of Sciences, Wrocław, Poland). The agar plates were incubated at 28 °C and examined daily under an optical microscope until amoebae were observed (up to 72 h). Genus discrimination was not conducted based on amoebae morphology. Then, two sets of new Petri dishes were prepared as described above. Small pieces of agar (approximately 5 mm × 5 mm) with groups of trophozoites were transferred onto new Petri dishes and placed top side down on the agar surface. To detect potentially pathogenic strains, one set was incubated at 37 °C and the second at 42 °C until amoebae were observed under the microscope (up to 72 h). The amoebae obtained from cultures incubated at 37 °C were washed with sterile PBS buffer (POL-AURA, Morąg, Poland), 1 mL per plate.

### 4.3. DNA Extraction from Soil and Cultures, and PCR Protocols

Two hundred microliters of PBS with washed amoebae from each agar plate were used for DNA extraction with the QIAamp DNA Mini Kit (Qiagen, Hilden, Germany). The Soil DNA Mini Kit (Syngen, Wrocław, Poland) was used for direct DNA extraction from soil samples (0.5 g of soil per isolation). DNA extractions were performed according to the manufacturer’s protocols. Five PCR protocols, were used to amplify the *SSU rRNA* gene fragments and detect the presence of FLAs in the examined samples (Table 2). Except for three primer pairs specific for a broad spectrum of FLAs, genus-specific primers were used for *Acanthamoeba* and the former genus *Hartmannella*, as they are common in the environment [12] and have been isolated earlier from environmental samples in Poland [41].

The protocols using HARTfor/HARTrev and Ami6F1/Ami9R primer sets were performed as previously described by the authors [43,45]. In the protocols using JDP1/JDP2, FLA-F/FLA-R, and AmeF977/AmeR153 primer pairs, annealing temperatures other than those in the original papers were used in the literature [15,16,17,18,19]. Thus, the temperatures used in this study were determined experimentally. PCR products were separated by electrophoresis in a 1.5% agarose gel (BioShop, Burlington, ON, Canada), stained with ethidium bromide (Sigma-Aldrich, St. Louis, MA, USA), and visualized under UV light.

### 4.4. Sequencing, Genotyping, and Phylogenetic Analysis

Both strands of all obtained PCR products were sequenced using the amplification primers (Table 2). The sequencing was performed at Macrogen Europe (Amsterdam, The Netherlands). The obtained sequences were compared with other homologous sequences deposited in the GenBank database using the Basic Local Alignment Search Tool (BLAST) at the National Center for Biotechnology Information. Separate alignments were performed for sequences of different genera of FLAs, using the MUSCLE algorithm (the MEGA12.0 software), which allowed for comparison to each other and to other homologous sequences from GenBank. The ends of the sequences in the alignments were trimmed to form blunt ends. The multiple alignment for the *Acanthamoeba* genus covered nucleotides corresponding to positions 720 to 1300 of the *Acanthamoeba* sp. genotype T4 sequence with GenBank accession number AY702983 (JDP1/JDP2 primer set). For the *Naegleria* genus, the multiple alignment covered nucleotides corresponding to positions 680–1343 (Ami6F1/Ami9R primer set), 756–1476 (FLA-F/FLA-R primer set), and 1277–1662 (AmeF977/AmeR1534 primer set) of the *N. gruberi* sequence with GenBank accession number OR769034. The phylogenetic trees were constructed using Mega 12 software, based on a multiple alignment and the neighbor-joining statistical method with the Kimura 2-parameter model, with 1000 bootstrap samples [47]. Different fragments of the same sequences from GenBank and the sequences obtained in this study were used to construct the trees based on the *18S rRNA* sequence of *Naegleria* spp. The fragment of the *SSU rRNA* gene obtained using the AmeF977/AmeR1534 primer set is not variable enough to construct a tree of clear topology. The sequences obtained with FLA-F/FLA-R and Ami6F1/Ami9R primers overlap to a large extent, leading to the construction of similar trees. FLA-F/FLA-R primers were more sensitive than Ami6F1/Ami9R, so the tree based on the sequences obtained with the first primer pair is presented (Figure 2). The results of the sequence comparison using BLAST and analysis of the phylogenetic trees’ topology were used to determine the genotype of *Acanthamoeba* and the species of *Naegleria* strains examined in this study. The genus or species of the remaining amoebae was determined based on the sequence comparison using BLAST. Phylogenetic trees for *V. vermiformis*, *P. bulliensis*, and *Paravahlkampfia* sp. were not constructed, as all obtained *V. vermiformis* sequences represent the same species and are all identical to those in the GenBank database. Sequences of the *Ptolemeba* and *Paravahlkampfia* genera deposited in GenBank are too few to use for phylogenetic analysis. The sequences analyzed in this study have been deposited in the GenBank database under accession numbers: PV867401-PV867419 (*Acanthamoeba* spp.), PV867462-PV867474 (*Naegleria* spp.), PV867476-PV867480 (*V. vermiformis*), PV867809 (*P. bulliensis*), and PV873343 (*Paravahlkampfia* sp.).

### 4.5. Statistical Analysis

Statistical analyses were performed using a chi-squared test to investigate the differences in the prevalence of various amoebae and the prevalence of amoebae in samples collected from specific zones in Babiogórski National Park. Statistical significance was defined as *p* < 0.05. The Statistica 13.3 software (StatSoft Inc., Tulsa, OK, USA) was used for the analysis.

## 5. Conclusions

The knowledge of FLA diversity in European soils is insufficient, and more studies regarding this topic are needed. This study revealed that the *Acanthamoeba* genus (T4 and T13 genotypes) dominates among FLAs occurring in soil from the Babiogórski National Park and its surroundings, followed by *Naegleria* spp. and *V. vermiformis*. It also reports the isolation of *Paravahlkampfia* sp., *P. bulliensis*, and *N. gruberi*, which are rarely detected in soil, as well as the first-time isolation of *N. galeacystis* from soil and *N. gruberi* and *V. vermiformis* from soil collected in Europe. Some of the detected FLAs are potentially pathogenic and may pose a health threat to humans. The universal primers used in this study do not reveal the full diversity of soil amoebae. It is necessary to design new, sensitive, and specific primers enabling the detection, species identification, and genotyping of a wide range of FLAs in soil.

## Figures and Tables

**Table 1:** The results of amoebae cultures at 42 °C, PCRs, and sequencing for the 24 strains that were grown at 28 °C and 37 °C.

Sample Number	Culture at 42 °C	PCR and Sequencing Results with Individual Primer Pairs				
		JDP1/JDP2	FLA-F/FLA-R	Ami6F1/Ami9R	AmeF977/ AmeR1534	HARTfor/ HARTrev
1.					*Naegleria* sp.	
2.	+	*Acanthamoeba* T13				*V*. *vermiformis*
4.		*Acanthamoeba* T4	*Paravahlkampfia* sp.			
6.	+	*Acanthamoeba* T4				
7.			*N*. *galeacystis*		*N*. *galeacystis*	
8.	+		*N*. *gruberi*	*N*. *gruberi*		
10.		*Acanthamoeba* T13	*N*. *galeacystis*		*N*. *galeacystis*	
11.			*N*. *gruberi*	*N*. *gruberi*	*Naegleria* sp.	
12.		*Acanthamoeba* T4				
15.	+	*Acanthamoeba* T4				
19.	+	*Acanthamoeba* T4	*V*. *vermiformis*		*V*. *vermiformis*	*V*. *vermiformis*
21.	+	*Acanthamoeba* T4				
23.		*Acanthamoeba* T13				
24.	+		*N*. *gruberi*	*N*. *gruberi*	*Naegleria* sp.	
27.		*Acanthamoeba* T4				
28.		*Acanthamoeba* T13			*P*. *bulliensis*	
29.	+	*Acanthamoeba* T4				
31.	+	*Acanthamoeba* T4				
32.	+	*Acanthamoeba* T13				*V*. *vermiformis*
33.	+	*Acanthamoeba* T13				
35.	+	*Acanthamoeba* T13				
37.		*Acanthamoeba* T4				
39.		*Acanthamoeba* T4				
40.		*Acanthamoeba* T4				

**Table 2:** Primer sets used in this study for FLA detection.

The Primer Set	Detecting FLAs	The Amplifying Fragment of the *18S rRNA* Gene and the Product Size	Hybridization Temperature	References
JDP1: 5′-GGCCCAGATCG- TTTACCGTGAA-3′ JDP2: 5′-TCTCACAAGCT- GCTAGGGAGTCA-3′	*Acanthamoeba* spp.	897–1358 bp of *A. castellani* sequence (U07400); 462 bp	55 °C	[16,42]
HARTfor: 5′-GGAGGGCAAGT- CTGGTGCC-3′ HARTrev: 5′-GCCCGGAGAGTCATCCATG-3′	Former genus *Hartmannella*	562–1095 bp of *V. vermiformis* sequence (EU137741); 534 bp	58 °C	[43]
FLA-F: 5′-CGCGGTAATTCC- AGCTCCAATAGC-3′ FLA-R: 5′-CAGGTTAAGGT- CTCGTTCGTTAAC-3′	All FLAs except *Balamuthia* and *Sappinia*	631–1614 bp of *A. castellani* sequence (U07400); 984 bp	55 °C	[16,44]
Ami6F1: 5′-CCAGCTCCAATAGCGTATATT-3′ Ami9R: 5′-GTTGAGTCGA- ATTAAGCCGC-3′	All amoebae	641–1468 bp of *A. castellani* sequence (U07400); 828 bp	55 °C	[45]
AmeF977: 5′-GATYAGATACCGTCGTAGTC-3′ AmeR1534: 5′-TCTAAGRGCAT- CACAGACCTG-3′	All amoebae	1179–1829 bp of *A. castellani* sequence (U07400); 651 bp	60 °C	[46]

## Data Availability

All data generated or analyzed in this study are included in this published article. The accession numbers of the DNA sequences obtained in this study are provided in the Materials and Methods Section and are available in the GenBank database (https://www.ncbi.nlm.nih.gov/genbank/, accessed on 19 May 2025).
